# Influenza and Pneumonia Mortality in 66 Large Cities in the United States in Years Surrounding the 1918 Pandemic

**DOI:** 10.1371/journal.pone.0023467

**Published:** 2011-08-19

**Authors:** Rodolfo Acuna-Soto, Cécile Viboud, Gerardo Chowell

**Affiliations:** 1 Departamento de Microbiologia y Parasitologia, Facultad de Medicina, Universidad Nacional Autónoma de México, México City, México; 2 Division of Epidemiology and Population Studies, Fogarty International Center, National Institutes of Health, Bethesda, Maryland, United States of America; 3 School of Human Evolution and Social Change, Mathematical, Computational & Modeling Sciences Center, Arizona State University, Tempe, Arizona, United States of America; University of Hong Kong, Hong Kong

## Abstract

The 1918 influenza pandemic was a major epidemiological event of the twentieth century resulting in at least twenty million deaths worldwide; however, despite its historical, epidemiological, and biological relevance, it remains poorly understood. Here we examine the relationship between annual pneumonia and influenza death rates in the pre-pandemic (1910–17) and pandemic (1918–20) periods and the scaling of mortality with latitude, longitude and population size, using data from 66 large cities of the United States. The mean pre-pandemic pneumonia death rates were highly associated with pneumonia death rates during the pandemic period (Spearman ρ = 0.64–0.72; P<0.001). By contrast, there was a weak correlation between pre-pandemic and pandemic influenza mortality rates. Pneumonia mortality rates partially explained influenza mortality rates in 1918 (ρ = 0.34, P = 0.005) but not during any other year. Pneumonia death counts followed a linear relationship with population size in all study years, suggesting that pneumonia death rates were homogeneous across the range of population sizes studied. By contrast, influenza death counts followed a power law relationship with a scaling exponent of ∼0.81 (95%CI: 0.71, 0.91) in 1918, suggesting that smaller cities experienced worst outcomes during the pandemic. A linear relationship was observed for all other years. Our study suggests that mortality associated with the 1918–20 influenza pandemic was in part predetermined by pre-pandemic pneumonia death rates in 66 large US cities, perhaps through the impact of the physical and social structure of each city. Smaller cities suffered a disproportionately high per capita influenza mortality burden than larger ones in 1918, while city size did not affect pneumonia mortality rates in the pre-pandemic and pandemic periods.

## Introduction

The “Spanish” influenza A/H1N1 pandemic of 1918 was a major epidemic event of the twentieth century. The influenza A/H1N1 virus infected approximately a third of the world's population and resulted in over 20 million deaths worldwide [1], [2]. Despite the relevance of this historical event for today's pandemic preparedness efforts and several decades of research focusing on the 1918 influenza virus, the biologic and epidemiologic factors accounting for the unusual severity of this pandemic remain unclear [3], [4], [5].

The role of influenza on seasonal increases in respiratory morbidity and mortality rates was not well understood when the 1918 pandemic struck, as the virus had not been identified. It was initially thought that Pfeiffer's bacillus, commonly known as *Haemophilus Influenzae*, which was identified in 1892, was the causative agent of influenza [6]. Moreover, the contribution of secondary and opportunistic bacterial infections, including *Streptococcus pneumoniae*, *Streptococcus pyogenes*, *Staphylococcus aureus*, *and H. Influenzae*, to disease severity was poorly understood [6].

It has been hypothesized that poor underlying health conditions, in part due to the occurrence of World War I, may have affected the severity of the 1918 pandemic. In particular, epidemiological studies have shown that geographical variation in baseline health conditions prior to the pandemic and the public health interventions implemented during the pandemic may partly explain the observed differences in pandemic mortality rates across cities, provinces, and countries [2], [7], [8]. The purpose of the present study is to test these relationships further by analyzing annual pneumonia and influenza mortality prior to and during the pandemic and explore the role of population and geographical factors in the 66 largest cities of the United States.

## Materials and Methods

### Historical Data

**Figure 1:**
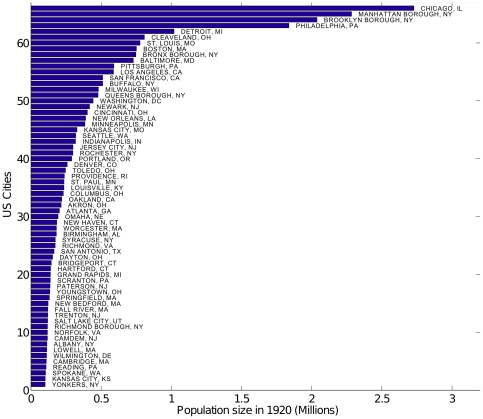
Population size of 66 larges US cities in 1920. The population size of the 66 largest US cities in 1920 shown in order of increasing population size.

We divided the 1910–1920 study period into a baseline pre-pandemic period (1910–17) and a pandemic period (1918–20), as several countries, including the US, experienced several waves of pandemic activity throughout 1918–1920 [7], [9]. The United States Department of Commerce Census Bureau provided vital statistics information in the largest cities of the US, defined by the Census as those with population larger than 100,000 in 1920 [10]. Annual influenza and pneumonia death rates per 100,000 inhabitants, latitude and longitude coordinates of population centers, and annual population estimates were compiled for the 66 listed largest cities [10], [11], after having excluded Memphis TN, Nashville, TN, Dallas, TX, and Houston, TX, due to incomplete records in 1910–1920 [10], [11]. In 1920, these 66 cities represented a total population of 26.9 millions, or 25.4% of the US population, with the largest city being Chicago, IL, with 2.7 million inhabitants (Figure 1). Total influenza and pneumonia death counts were estimated by multiplying mortality rates by population size for each city and year.

### Excess mortality and predictive power of baseline pneumonia and influenza death rates

We calculated excess death counts associated with the pandemic as the death counts observed during pandemic years minus the average death counts for pre-pandemic years (1910–1917). We also calculated excess death rates per 100,000 inhabitants using our estimate of population size of each city for the corresponding pandemic year. We calculated Spearman non-parametric correlation coefficients between the mean pneumonia and influenza death rates during pre-pandemic and pandemic years across the 66 US cities.

### The relationship between pneumonia and influenza mortality and population size

City size has been found to follow a simple scaling relationship with measures of energy consumption, economic activity, demographics, innovation, patterns of human behavior, infectious disease burden, among others [12]. Next, we characterized the functional relationship between population size and annual pneumonia and influenza deaths in US cities for each year between 1910–1920, using methods previously applied to explore spatial heterogeneity in influenza disease rates [13], [14]. A power-law function of population size follows if D α N^α^, where D denotes number of deaths per year, N indicates city-level population size, and α is determined by the proportionality of population to mortality rate [12], [13]. For α = 1.0, deaths are exactly proportional to population size (i.e. there is no heterogeneity as death rates are constant across cities); α<1 indicates that low population areas experience higher death rates, while the opposite is true for α>1.

## Results

### Time series of annual influenza and pneumonia death counts in 66 US cities

**Figure 2:**
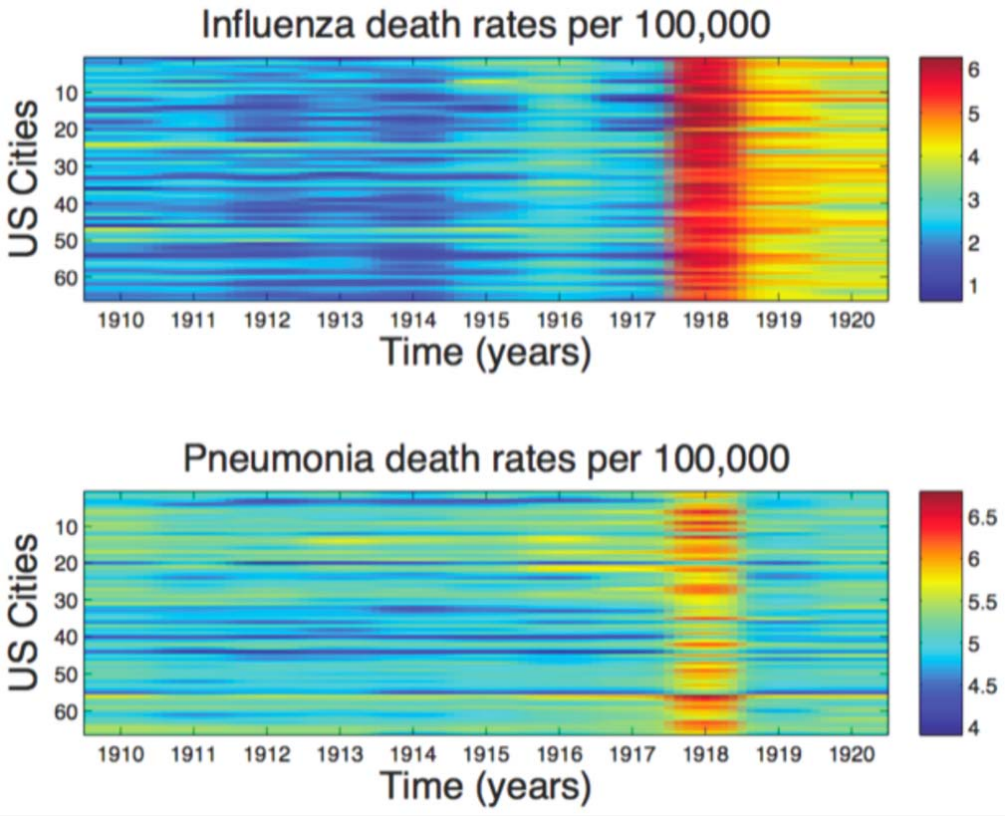
Influenza and pneumonia death rates 1910–1920 in 66 large US cities. Annual influenza and pneumonia death rates (log scaled) in each of the 66 US cities in order of increasing population size, 1910–1920.

A list of the 66 large US cities studied, latitude and longitude coordinates, and baseline demographic information are provided in Table 1. Figure 2 shows the time series of annual influenza and pneumonia death rates during 1910–20 in each of these cities in the order of increasing population size. There was no correlation between city size and latitude (Spearman ρ = −0.06, P = 0.62) or longitude coordinates (Spearman ρ = 0.22, P = 0.07). In the baseline pre-pandemic period (1910–17), the mean death rate per year due to pneumonia ranged between 64 and 266 deaths per 100,000 across the 66 cities. During the same period, the average annual influenza death rate ranged from 3 to 38 deaths per 100,000. In 1918, however, the average influenza death rate increased by a factor of 10 to 129-fold over the baseline. In contrast, the average pneumonia death rate only increased by a factor of 1.2 to 3.6-fold. In 1918, excess mortality due to influenza and pneumonia was 64,139 and 48,244 deaths, respectively, representing an excess pneumonia and influenza death rate of 437 per 100,000.

**Table 1:** Latitude and longitude coordinates, population size, and mean baseline pneumonia and influenza death rates for 66 large US reporting cities (1910–1920) with 100, 000 or more inhabitants [10].

66 US Cities	Latitude (°N)	Longitude (°W)	Population size (1920)	Mean baseline (1910–1917) pneumonia death rates (per 100,000)	Mean baseline (1910–1917) influenza death rates (per 100,000)
YONKERS, NY	40.917	73.883	101236	146.14	6.55
KANSAS CITY, KS	39.100	94.617	102080	151.30	20.78
SPOKANE, WA	47.667	117.433	104439	89.55	11.14
READING, PA	40.333	75.917	108231	112.81	13.92
CAMBRIDGE, MA	42.233	71.800	109944	160.69	7.36
WILMINGTON, DE	39.733	75.533	111248	191.00	15.06
LOWELL, MA	42.633	71.300	113092	165.03	13.07
ALBANY, NY	42.667	73.750	113920	148.46	21.18
CAMDEM, NJ	39.883	75.117	117430	201.87	12.52
NORFOLK, VA	36.833	76.283	117605	126.16	14.90
RICHMOND BOROUGH, NY	40.783	73.967	118105	164.38	6.89
SALT LAKE CITY, UT	40.767	111.900	119272	103.26	6.62
TRENTON, NJ	40.217	74.733	120446	206.37	9.83
FALL RIVER, MA	41.701	71.155	120546	229.13	7.32
NEW BEDFORD, MA	41.633	70.933	122482	177.94	6.49
SPRINGFIELD, MA	42.100	72.567	131702	134.05	13.62
YOUNGSTOWN, OH	41.083	80.633	134716	201.13	6.00
PATERSON, NJ	40.917	74.167	136405	162.58	7.72
SCRANTON, PA	41.400	75.650	138191	212.86	9.52
GRAND RAPIDS, MI	42.967	85.667	138822	75.13	4.50
HARTFORD, CT	41.750	72.683	140051	199.81	13.72
BRIDGEPORT, CT	41.167	73.200	145693	180.23	10.38
DAYTON, OH	39.750	84.183	154413	116.23	10.09
SAN ANTONIO, TX	29.383	98.550	164714	93.12	37.50
RICHMOND, VA	37.550	77.483	173007	156.94	18.54
SYRACUSE, NY	43.033	76.133	173767	129.56	5.63
BIRMINGHAM, AL	33.500	86.833	181249	168.44	16.59
WORCESTER, MA	42.250	71.800	181494	173.08	6.62
NEW HAVEN, CT	41.317	72.917	184027	193.04	15.06
OMAHA, NE	41.250	95.933	193423	127.41	9.86
ATLANTA, GA	33.750	84.383	202902	156.28	15.48
AKRON, OH	41.067	81.517	215355	63.82	8.85
OAKLAND, CA	37.800	122.267	219665	86.66	4.55
COLUMBUS, OH	40.000	83.017	230907	116.68	12.75
LOUISVILLE, KY	38.250	85.767	235299	146.60	17.81
ST. PAUL, MN	44.933	93.083	235726	94.44	6.01
PROVIDENCE, RI	41.833	71.400	238279	177.97	12.81
TOLEDO, OH	41.650	83.550	246675	101.07	11.12
DENVER, CO	39.750	105.000	258712	128.03	8.24
PORTLAND, OR	45.517	122.683	290478	64.63	7.13
ROCHESTER, NY	43.150	77.600	298910	120.21	5.89
JERSEY CITY, NJ	40.717	74.067	299655	184.44	7.15
INDIANAPOLIS, IN	39.767	86.167	318007	125.13	11.41
SEATTLE, WA	47.617	122.333	319284	66.34	5.58
KANSAS CITY, MO	39.100	94.583	328326	128.04	13.34
MINNEAPOLIS, MN	44.983	93.233	384660	103.12	4.71
NEW ORLEANS, LA	29.950	90.067	389688	154.91	32.99
CINCINNATI, OH	39.133	84.500	401971	138.76	18.03
NEWARK, NJ	40.733	74.167	417978	153.49	9.83
WASHINGTON, DC	38.883	77.033	443056	134.20	30.47
QUEENS BOROUGH, NY	40.783	73.967	478585	130.88	6.29
MILWAUKEE, WI	43.033	87.917	480894	122.31	8.83
BUFFALO, NY	42.917	78.833	511053	143.93	5.99
SAN FRANCISCO, CA	37.783	122.433	511300	117.27	2.89
LOS ANGELES, CA	34.050	118.250	588328	70.11	6.59
PITTSBURGH, PA	40.450	79.950	591033	265.85	13.23
BALTIMORE, MD	39.300	76.633	729506	197.63	12.52
BRONX BOROUGH, NY	40.783	73.967	747520	125.77	6.29
BOSTON, MA	42.350	71.083	751252	199.89	5.77
ST. LOUIS, MO	38.583	90.200	777320	152.31	16.19
CLEAVELAND, OH	41.467	81.617	808273	116.27	8.81
DETROIT, MI	42.333	83.050	1019289	113.61	4.62
PHILADELPHIA, PA	39.950	75.167	1837924	155.48	13.48
BROOKLYN BOROUGH, NY	40.783	73.967	2038118	186.75	8.14
MANHATTAN BOROUGH, NY	40.783	73.967	2284103	219.47	5.78
CHICAGO, IL	41.833	87.617	2728022	167.37	7.05

### Pandemic predictive power of baseline pneumonia and influenza mortality

**Figure 3:**
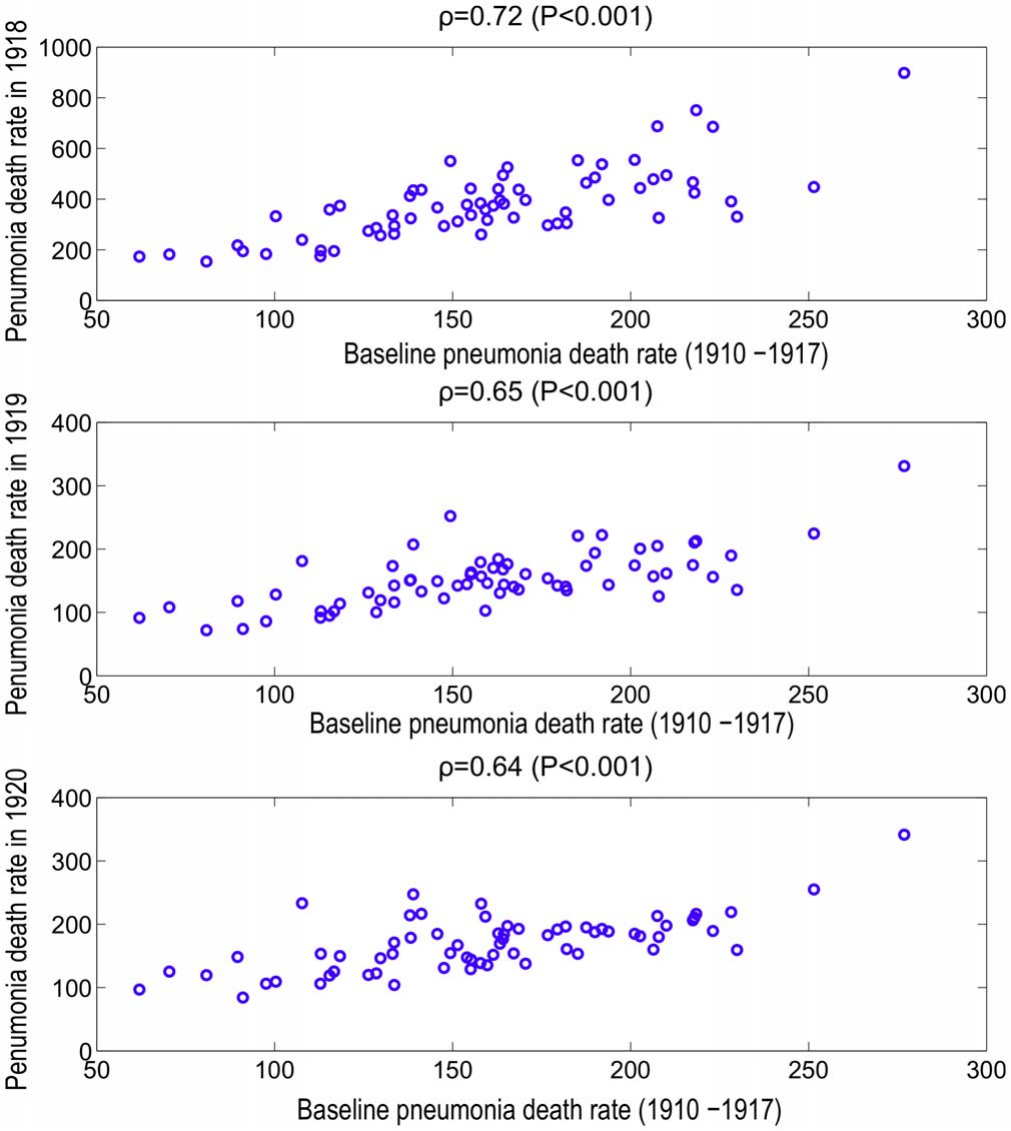
Correlation between pneumonia death rate before pandemic influenza and pneumonia death rate during pandemic influenza in 66 large US cities. The correlation between the mean baseline pneumonia death rate during 1910–1917 and the pneumonia death rate in 1918, 1919 and 1920 across the 66 US cities.

**Figure 4:**
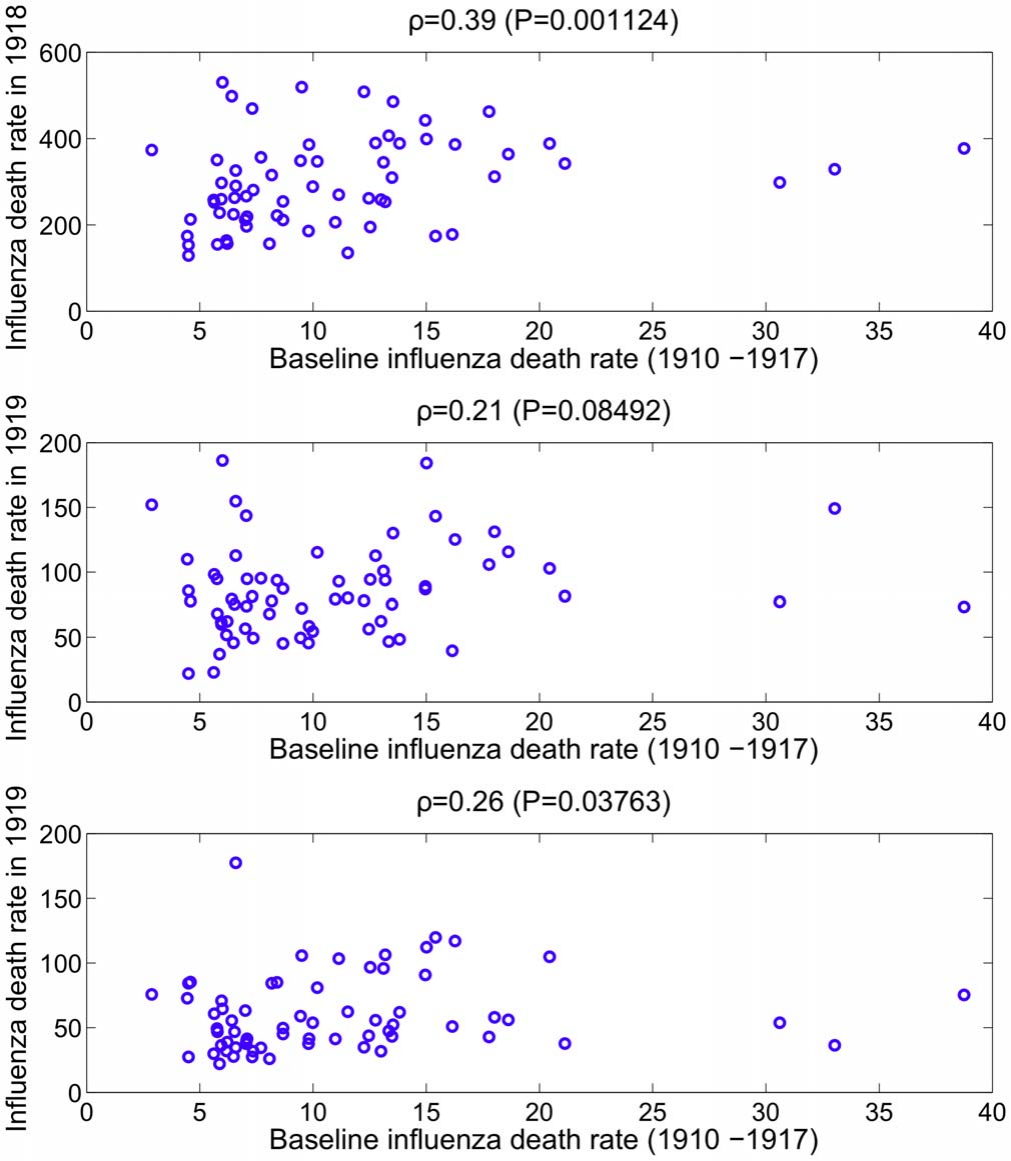
Correlation between influenza mortality rate before and during pandemic influenza in 66 large US cities. The correlation between the mean baseline influenza death rate during 1910–1917 and the influenza death rate in 1918, 1919 and 1920 across the 66 US cities.

We found a significant correlation between the mean baseline pneumonia death rate during 1910–1917 in the 66 US cities and the pneumonia death rate during each of the pandemic years from 1918 to 1920 (Spearman ρ = 0.64–0.72; P<0.001) (Figure 3). By contrast, mean baseline influenza death rates were only weakly correlated with influenza death rate during the pandemic period (Figure 4). Furthermore, pneumonia death rates partially explained influenza death rates in 1918 (Spearman ρ = 0.34, P = 0.005), but not during any of the other years of the study period (Table 2). Baseline influenza mortality rates were weakly correlated with pneumonia death rates in 1918 and vice versa (Spearman ρ = 0.26–0.40, P<0.03). As a sensitivity analysis, we repeated the analysis by aggregating death rates for the 5 boroughs of New York City. Our results were qualitatively similar following this change.

**Table 2:** Spearman correlation coefficient (and corresponding P value) between pneumonia and influenza mortality rates in baseline years (1910–17) and pandemic years (1918–20) in 66 US cities.

	Baseline Influenza 1910–17	Baseline Pneumonia 1910–17	Pandemic Influenza 1918	Pneumonia 1918	Pandemic Influenza 1918–20	Pneumonia 1918–20
Baseline Influenza 1910–17	1.0	0.21 (0.1)	0.39 (0.001)	0.26 (0.03)	0.42 (0.0005)	0.29 (0.02)
Baseline Pneumonia 1910–17	0.21 (0.1)	1.0	0.4 (<0.001)	0.8 (<0.0001)	0.3 (0.02)	0.83 (<0.0001)
Pandemic Influenza 1918	0.39 (0.001)	0.4 (<0.001)	1.0	0.34 (0.005)	0.92 (<0.0001)	0.33 (0.006)
Pneumonia 1918	0.26 (0.03)	0.8 (<0.0001)	0.34 (0.005)	1.0	0.19 (0.13)	0.98 (<0.0001)
Pandemic Influenza 1918–20	0.42 (0.0005)	0.3 (0.02)	0.92 (<0.0001	0.19 (0.13)	1.0	0.21 (0.09)
Pneumonia 1918–20	0.29 (0.02)	0.83 (<0.0001)	0.33 (0.006)	0.98 (<0.0001)	0.21 (0.09)	1.0

### Relationship between pneumonia and influenza mortality and population size

**Figure 5:**
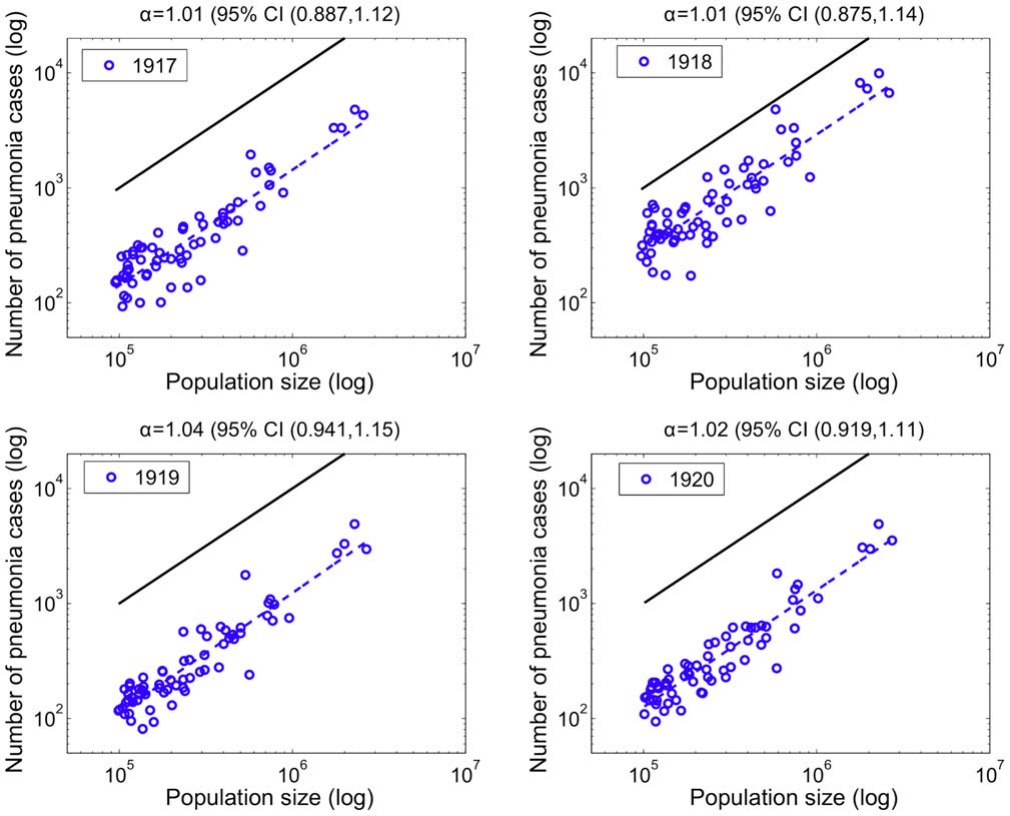
Relationship between the number of pneumonia deaths and population size for 66 US cities. Relationship between the total number of pneumonia deaths and population size for the 66 US cities. The dashed blue line represents the best linear fit to the data in log-log scale. A solid black line representing a slope of one is shown as a reference to illustrate the expected relationship if pneumonia mortality rates did not vary with population size. The slope of the observed data is ‘linear’ (not significantly different than one) for all years suggesting invariant death rates before and during the 1918 infuenza pandemic.

**Figure 6:**
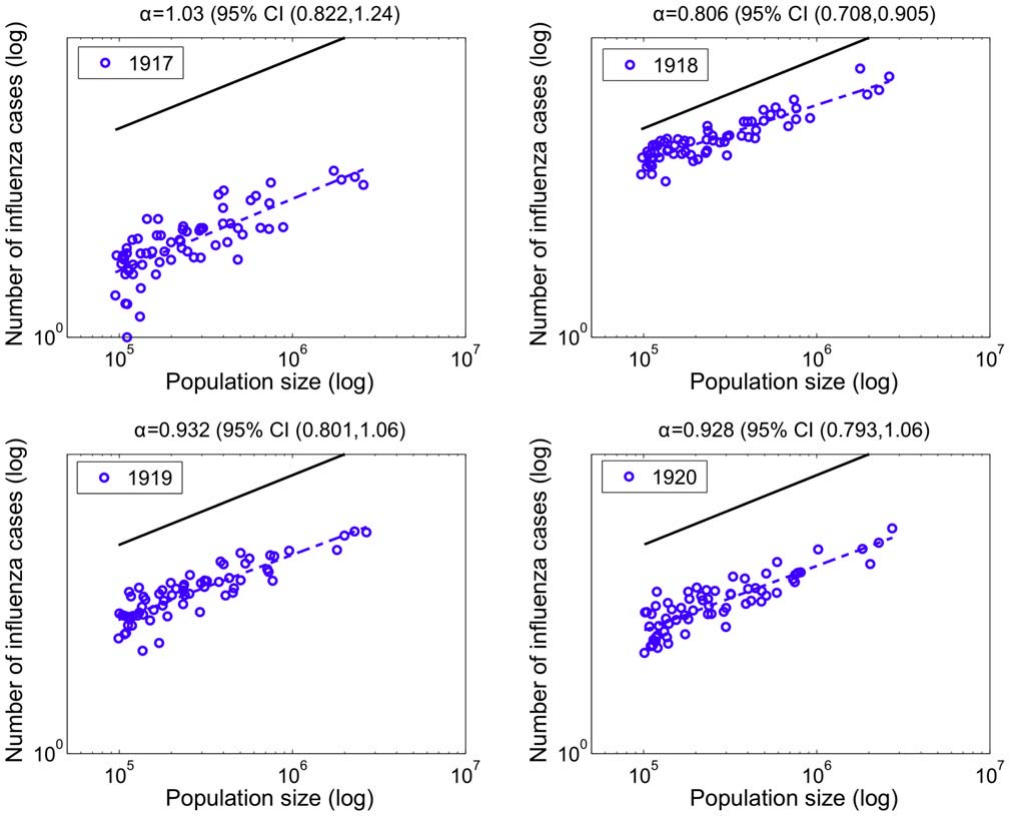
Relationship between the number of influenza deaths and population size for 66 US cities. Relationship between the total number of influenza deaths and population size for the 66 US cities. The dashed blue line represents the best linear fit to the data in log-log scale. A solid black line representing a slope of one is shown as a reference to illustrate the expected relationship if influenza mortality rates did not vary with population size. The slope of the observed data is ‘linear’ for all years (invariant death rates across cities) except for a slope less than one for year 1918 suggesting that less populous cities were more heavily affected during the 1918 influenza pandemic.

Pneumonia death counts followed a linear relationship with population size in all pre-pandemic and pandemic years, suggesting that population size did not affect pneumonia death rates in the 66 large US cities (α exponent not statistically significantly different from 1.0, Figure 5). In contrast, influenza death counts followed a power law with population size in 1918 with a scaling exponent *α* of ∼0.81 (95%CI: 0.71, 0.91), indicating that smaller cities systematically experienced higher death rates (Figure 6). Scaling exponents *α* for influenza death counts were not statistically significantly different from 1.0 in 1919 and 1920 (Figure 6) or in the pre-pandemic period (1910–1917). The scaling exponent using pneumonia death counts for the aggregated period 1918–1920 was not significantly different to those exponents obtained for individual years. Using influenza death counts, the corresponding scaling exponent for the aggregated period 1918–1920 was significantly less than one (α = 0.85, 95%CI: 0.76–0.93). Overall, our power law analysis suggests that baseline pneumonia and influenza death rates were independent of population size, but population size was associated with influenza death rates during the main pandemic wave in 1918.

### Relationship between excess pneumonia and influenza death rates, pre-pandemic death rates, and population size

The correlation between excess influenza death rates and pre-pandemic death rates was weakly but statistically significant in 1918 (Spearman ρ = 0.28, P = 0.03) whereas no significant trend was found in 1919 and 1920 (P>0.4). We found a moderate and significant correlation between population size and excess influenza death rates in 1918 across US cities (Spearman ρ = −0.45, P = 0.0001), but this correlation was not significant in 1919 and 1920.

For 1918, excess pneumonia death rates were not significantly correlated with the baseline pre-pandemic pneumonia death rates or population size. In 1919 and 1920, a number of cities experienced negative excess death rates.

## Discussion

The main finding of this study is the existence of a correlation between baseline pre-pandemic pneumonia mortality rates in 66 US cities and influenza-specific mortality rates during the pandemic years of 1918–1920. It is interesting that cities of all sizes experienced high mortality rates in the pandemic period, provided they had high pre-pandemic mortality levels. Influenza and pneumonia mortality rates in each individual year between 1910 and 1917 were strongly correlated with mortality rates in 1918, indicating that this was not a spurious association.

The statistical associations between influenza and pneumonia death rates may represent a strong biological interaction between influenza viruses and respiratory bacterial pathogens associated with pneumonia. A possible synergistic association between the influenza virus and bacterial pneumonia has been suspected for a long time in the context of the 1918 pandemic influenza [15]. Over the last few years, a series of reports have revealed the major role of bacterial pneumonia in the pathology of the 1918 pandemic virus. Lung tissue sections obtained from a long series of autopsies indicate that most influenza-related fatalities in 1918 were associated with secondary bacterial pneumonia [16], [17]. Also, recent experimental evidence indicates that influenza infection leads to increased susceptibility to subsequent bacterial infection [18], [19], [20], [21].

In 1924 Tomanek and Wilson concluded that pneumonia mortality rates were stable in US cities from 1910 to 1920, except for 1918, using the same data as in our study. The authors commented that pneumonia is the terminal phase of a diverse group of diseases, resulting in large uncertainty in the diagnosis of “pneumonia” and “influenza” [22]. Eighty-six years after the Tomanek and Wilson publication, this issue remains topical, and we question the true meaning of “influenza” and “pneumonia” as defined by the official vital statistics databases. This aspect of the influenza epidemiology has been troubling researchers for years [15], [23] and could explain the lack of association between baseline influenza death rates and pandemic death rates in our study.

Our analysis of death rates and population sizes suggest that smaller cities suffered a disproportionately large mortality burden due to influenza in 1918, as compared with larger cities. These results could be explained by differences in health care, socioeconomic status, or baseline health status between cities. Our findings echo those of a study of the 1918 influenza pandemic in rural areas of England and Wales, where less populated areas experienced higher influenza death rates than more populous ones [13]. We note that the scaling exponents tend to be lower in England and Wales than in the US, and in contrast to the US, there was no population effect in cities of England and Wales. It is possible that levels of geographical aggregation and population mixing, which differ between these two studies, affect the relationship between influenza-related death rate and population size. It is also important to note that in our US study, the per capita risk of pneumonia mortality was similar in cities of all sizes and in all study years, suggesting that heterogeneity in influenza disease rates may only be identified during extreme events (pandemics), and with specific indicators (influenza-specific deaths).

The role of baseline socio-demographic or geographical factors on the health impact of the 1918 influenza pandemic remains debated. Specifically, in a study of four British cities, no association was found between influenza attack rates and residential crowding [24]. Similar results were obtained by a study of the 1918–1919 influenza pandemic that considered measures of population density, residential crowding, and pre-pandemic infant mortality rates across 305 administrative areas of England and Wales [13]. In fact, this study found urbanization to be the most significant predictor of death rates, with cities and towns experiencing approximately 30–40% higher death rates than rural areas during both pandemic waves. In the US, influenza-related mortality rates in 1918 have been moderately associated with population density and baseline mortality rates [7], [25]. Other studies, by contrast, have reported a strong association between socio-demographic characteristics and 1918–1919 pandemic mortality rates, including per capita income and indicators of wealth, like apartment size [2], [26]. More recently, an association between high disease rates and low population size was reported in Mexico during the 2009 pandemic [14] reminiscent of our results for the 66 large cities in the US. Differences in geographic and temporal resolution could potentially explain these conflicting results [13].

There are some caveats associated with the nature of the data used in our study. First, differential mortality reporting and death coding practices, both spatial and temporally, could be potential sources of bias in our study. During the time period covered in this study mortality registration was functional in specific areas, mainly in large metropolitan areas, while registration in rural areas were in different developmental stages. The process of incorporation of vital statistics from states and cities was progressive and restricted to those areas with laws and ordinances requiring mandatory registration of deaths with a standard medical certificate. In the opinion of the Director of the Bureau of Census the data quality was satisfactory as early as in 1904 [27]. In this study, we assumed that reporting and coding of death certificates were homogeneous across large US Cities and over the course of the pandemic, in the absence of specific information on reporting quality. In addition, the fact that low population size was associated with high influenza death rates in 1918 suggesting that if anything, reporting and coding of influenza deaths was stronger in the smaller cities, where influenza awareness may have been less common. Second, our analyses did not employ age-standardized mortality rates, which are best suited to geographical comparisons where estimates of age-specific deaths and population sizes are available. This is particularly important because children have been identified as major players of influenza spread and because the 1918–1919 influenza pandemic was characterized by a disproportionate large mortality impact among young adults [9], [28], [29]. Nevertheless, a prior study relying on highly resolved spatiotemporal data of the 1918 influenza pandemic in England and Wales did not find a significant correlation between the proportion of children or young adults and the geographical variations in influenza transmissibility and death rates. Third, we used annual deaths where pneumonia or influenza is listed as the underlying cause of death, which are crude proxies of influenza-related burden. A more precise approach relies on the estimation of “excess” deaths above a seasonal baseline mortality curve that is calibrated using weekly or monthly mortality data [30]. Despite using a crude approach to determining excess mortality, our estimates of excess mortality rates are very much in line with those using more sophisticated approaches. Specifically, our mean excess mortality rate of 0.44% based on annual data from 66 cities is very similar to weekly-based estimates for New –York City [9] and Copenhagen [28]. Fourth, we did not have access to data on social or medical factors that may confound the association between baseline pre-pandemic pneumonia death rates, population sizes, and pandemic death rates, including crowding, prevalence of chronic respiratory conditions (eg, tuberculosis), healthcare quality, and socio-economic status. Finally, it is also worth noting that our study concentrated on 66 large US cities, because we did not have access to data for smaller cities and rural areas. Such data would have captured more fully the spatial heterogeneity of pneumonia and influenza mortality in the US.

A better understanding of the biological and epidemiological processes that influence the interaction between influenza viruses and the bacteria associated with pneumonia (and pneumonia-related mortality) can provide the basis for better influenza treatment and prevention programs. This pathogen-interaction issue is not only a matter of historical interest. Today, the association between influenza and bacterial infection remains a cause of elevated morbidity and mortality during pandemic [16], [17] and inter-pandemic periods [31]. Our study suggests the presence of a strong interaction during the 1918 “Spanish” influenza pandemic.
